# Quantum gates by periodic driving

**DOI:** 10.1038/srep22077

**Published:** 2016-02-25

**Authors:** Z. C. Shi, W. Wang, X. X. Yi

**Affiliations:** 1 Center for Quantum Sciences and School of Physics, Northeast Normal University, Changchun 130024, China; 2 School of Physics and Optoelectronic Technology Dalian University of Technology, Dalian 116024, China

## Abstract

Topological quantum computation has been extensively studied in the past decades due to its robustness against decoherence. One way to realize the topological quantum computation is by adiabatic evolutions—it requires relatively long time to complete a gate, so the speed of quantum computation slows down. In this work, we present a method to realize single qubit quantum gates by periodic driving. Compared to adiabatic evolution, the single qubit gates can be realized at a fixed time much shorter than that by adiabatic evolution. The driving fields can be sinusoidal or square-well field. With the sinusoidal driving field, we derive an expression for the total operation time in the high-frequency limit, and an exact analytical expression for the evolution operator without any approximations is given for the square well driving. This study suggests that the period driving could provide us with a new direction in regulations of the operation time in topological quantum computation.

As a promising avenue to deal with decoherence, topological quantum computations^1^,^2^,^3^,^4^,^5^,^6^,^7^,^8^ employ two-dimensional quasiparticles called anyons, whose world lines cross over one another to form braids in a three-dimensional spacetime. Information encoded in the anyons is robust against local perturbations, due to the topological nature of anyons, and braiding operation^2^ can be exploited to construct fault-tolerant quantum computation^9^. Majorana fermions, the simplest example of non-Abelian anyons, are predicted to exist in fractional quantum Hall systems^10^, topological insulators^11^,^12^, solid state systems^13^, and semiconductor-superconductor hybrid systems^14^,^15^,^16^. More recently, the signatures of Majorana fermions have also been observed in experiments^17^,^18^,^19^,^20^,^21^, which gives rise to an opportunity to encode a qubit by Majorana fermions in these materials.

The adiabatic evolution has been widely applied to preparation and manipulation of Majorana fermions^22^,^23^,^24^,^25^. In particular, it has been shown that topological quantum information processing becomes possible in the one-dimensional network^25^ by adiabatically controlling the locally tunable gates which affect the chemical potential over a finite length of the topological superconductor (TS) wire. The main idea of adiabatic computation is that, design a Hamiltonian *H*_1_ whose ground state is the target state 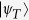 while the ground state 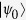 of Hamiltonian *H*_0_ is easily to prepared. Assume that there exists a quantum system described by the following Hamiltonian,


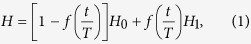


where *f*(*t*) is a slowly varying function of time *t* with *f*(0) = 0 and *f*(1) =1. According to the adiabatic theorem, the quantum system evolves adiabatically from the initial (ground) state 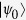 to the target (ground) state 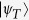 at time *t *= *T*, solving the problem associated with the target state 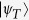. The price one shall pay in adiabatic computation is the long evolution time required by adiabatic condition.

Since a quantum task is often accomplished by a sequence of quantum operations rather than a single quantum operation^23^,^24^,^25^,^26^,^27^,^28^,^29^, the total operation time increases linearly with the number of quantum operations. On the other hand, with respect to the limit of the coherence time in quantum systems, long operation time is not favorable for quantum computation, even for topological quantum computation which is robust against perturbations. Hence it is not suitable for implementing quantum operations by adiabatic evolution if the coherence time of quantum systems are short. This gives rise to a question that are there any other methods to achieve the goals better than adiabatic passage?

The time-periodic driving systems have been extensively studied in the past few years. Several work^30^,^31^,^32^,^33^,^34^,^35^,^36^,^37^,^38^,^39^,^40^,^41^ have shown that topological properties of system can be changed by time-periodic driving (e.g., the existence of Floquet topological insulators or Floquet Majorana fermions). Recently, the Floquet Majorana fermions are realized by periodic driving fields in the system of coupled quantum dots proximity to a *s*-wave superconductor^42^. More recently, it has been proposed to achieve the direct coupling between the topological and conventional qubits by periodic driving fields^43^. In this work we explore the possibility to regulate the total operation time by periodic driving. Of course, this method can also be extended to the other quantum systems.

## Results

**Figure 1:**
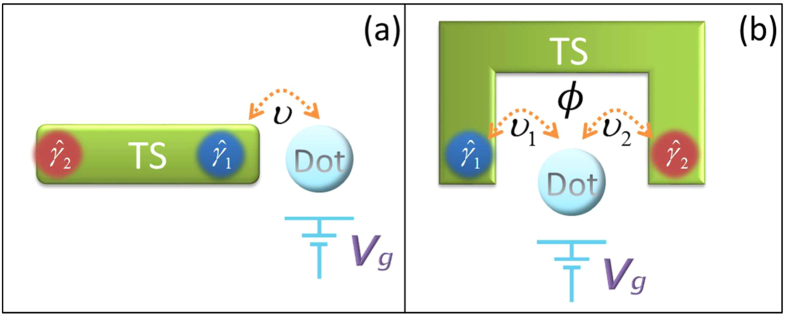
The schematic setup for realizing the operation *P*_1_.

The physical model of interest consists of a quantum dot coupling to a TS nanowire, as shown in Fig. 1(a). In the magnetic field, by proximity coupling to a superconductor, one can prepare Majorana modes in the nanowire with strong spin-orbit interaction in the topological phase^15^,^16^,^44^,^45^. Then the effective Hamiltonian (in the low-energy limit) for the quantum dot coupling to the Majorana mode reads^46^


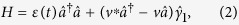


where *a* (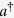) is the annihilation (creation) operator for electron in the quantum dot and the on-site energy 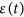 of the quantum dot can be controlled by the gate voltage *V*_*g*_. *v* denotes the tunnel coupling between the quantum dot and the Majorana mode 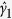. Without loss of generality we assume *v* is an real number and take all physical parameters in units of *v*. Since the Majorana mode 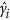 (*i* = 1, 2) is Hermitian (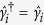 and 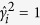), we cannot use the number operator 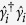 to count the population of Majorana mode. Nevertheless, two Majorana modes can be combined to generate one ordinary fermion, e.g., 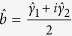. One can adopt the number operator 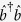 of the ordinary fermion to count Majorana modes.

Due to the conservation of total parity, defined by the electron in quantum dot and the ordinary fermion formed by Majorana modes, the Hamiltonian is block diagonal in the basis spanned by 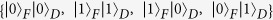,


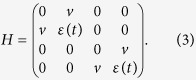


We have used 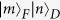 to describe system state, where *m* is the number of ordinary fermion formed by the Majorana modes and *n* is the number of electron in the quantum dot. It has been suggested^46^ that by adiabatically changing the values of the on-site energy 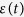 from 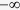 to 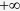, one can realize the operation *P*_1_ which denotes the population inversion in the ordinary fermion formed by the Majorana modes, i.e.,


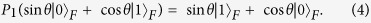


Actually, the operation *P*_1_ is equivalent to the action of operator 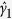, i.e., 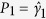.

**Figure 2:**
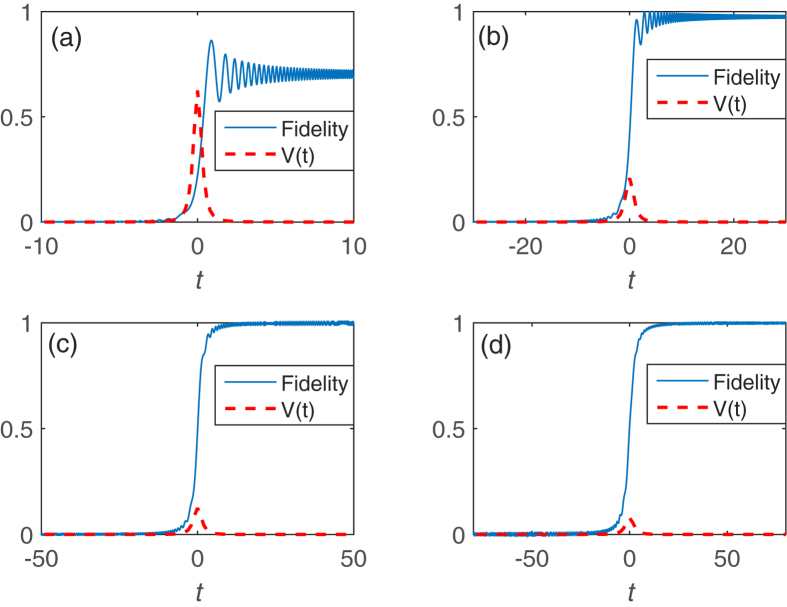
Fidelity and *V* as a function of time in the realization of the operation *P*_1_. Here the fidelity is defined as 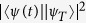. The initial state is 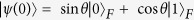 and the target state is 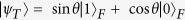, 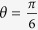. We have set the on-site energy of the quantum dot to increase linearly with time, e.g., 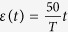. The operation time is 2*T* and the final value of 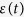 is 50 during the time evolution. All parameters are in units of the tunnel coupling *v*. (**a**) *T* = 10. (**b**) *T* = 30. (**c**) *T* = 50. (**d**) *T* = 80. Large *T* means small changing rate of the on-site energy 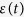. One can find that the time to obtain the operation *P*_1_ is about 2*T* = 100.

We first present the results of realizing the operation *P*_1_ by adiabatic evolution. Figure 2 shows different dynamical behaviors with different changing rates of the on-site energy 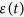. Note that we employ 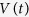 defined by 
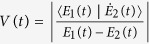
 to quantify the adiabatic condition, where 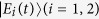 is the instantaneous eigenstate (the corresponding eigenvalue is denoted by 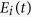) of the Hamiltonian in the even (or odd) subspace. It can be observed in Fig. 2(a) that the operation *P*_1_ cannot be achieved perfectly since the changing rate of the on-site energy 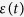 is too fast to satisfy the adiabatic condition very well, reflecting in the aspect that 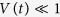 cannot be fulfilled all the time. In order to successfully realize the operation *P*_1_, the changing rate of on-site energy 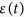 should be slow, which increase the operation time (cf. Fig. 2(c,d)). Considering that the operation *P*_1_ is necessary to implement single qubit rotations or non-Abelian operations, the total operation time would be proportional to the number of operation *P*_1_.

Besides, noting that the Majorana based qubits may be sensitive to the decoherence induced by electron tunnel coupling to an environment (e.g., leads)^47^,^48^,^49^,^50^, the adiabatic operation may have no use in minimizing the influence of the decoherence. Recently, the adiabatic evolution can be speeded up by the short-cut scheme and it has been used in the non-Abelian braiding with Y-junction structure^51^, but it needs coupling between distinct Majorana modes which seems not very easy in experiment. We will demonstrate in the following that the operation time can be regulated by periodic driving.

### Sinusoidal driving

**Figure 3:**
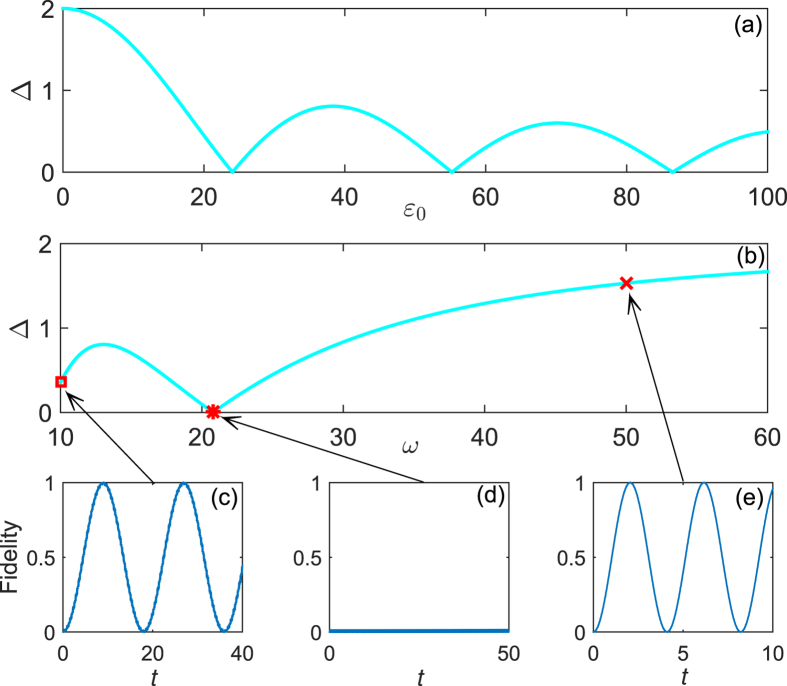
The quasi-energies gap versus (**a**) the amplitude 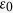 when 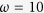, (**b**) the frequency 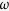 when 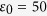. The quasi-energies gap approaches 2 when the driving frequency tends to 60 in panel (**b**). After that the gap increases slowly with the driving frequency. The system dynamics by sinusoidal driving with (**c**) 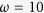, (**d**) 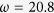, and (**e**) 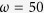. All parameters are in units of the tunnel coupling 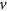.

We first consider a periodic modulation of the on-site energy for the quantum dot, where the on-site energy takes the sinusoidal form 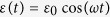. The modulation can be generated by waveform generator experimentally and we would also call 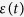 as driving field hereafter. For a periodic driving system, it is instructive to make definite on the time-scale during evolution. Since the Floquet state 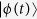 has the same period with the driving field (see Methods), it affects the system dynamics on short time-scale in the high-frequency limit. What really affects the long time-scale of the system dynamics is the gap of quasi-energies. Therefore, it is helpful to estimate the gap of quasi-energies in the periodic driving system. In order to obtain an approximate expression for quasi-energies, we solve the time-dependent Schrödinger equation by standard perturbation theory, where the tunnel term is regarded as a perturbation. After some algebra, the quasi-energies gap is given by 
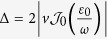
 (see Methods). Figure 3(a,b) demonstrate the relation between the parameters of driving field and the quasi-energies gap, while Fig. 3(c–e) show the time evolution of the system with different quasi-energies gaps. Obviously, the time-scale of system dynamics gets short with the increasing of the quasi-energies gap. An inspection of Fig. 3(a) also shows that the operation time varies with the decreasing of the amplitude of driving field at a fixed high frequency. Therefore, we can control the operation time in the periodic driving system by properly regulating the frequency and amplitude of driving field. Interestingly, there exists a special case where the quasi-energies vanish at a designated amplitude and frequency of driving field (namely two quasi-energies are degenerate), which is known as the coherent destruction of tunneling^52^,^53^. As a consequence the state is highly localized, and it is not valid to achieve the operation 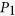, as shown in Fig. 3(d).

Taken 
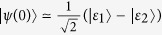
 as the initial state, where 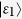 and 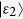 are given in Methods, the time evolution of periodic driving system reads


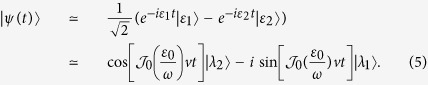


We find that the total operation time for realizing the operation 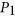 approximately equals


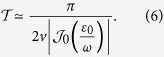


**Figure 4:**
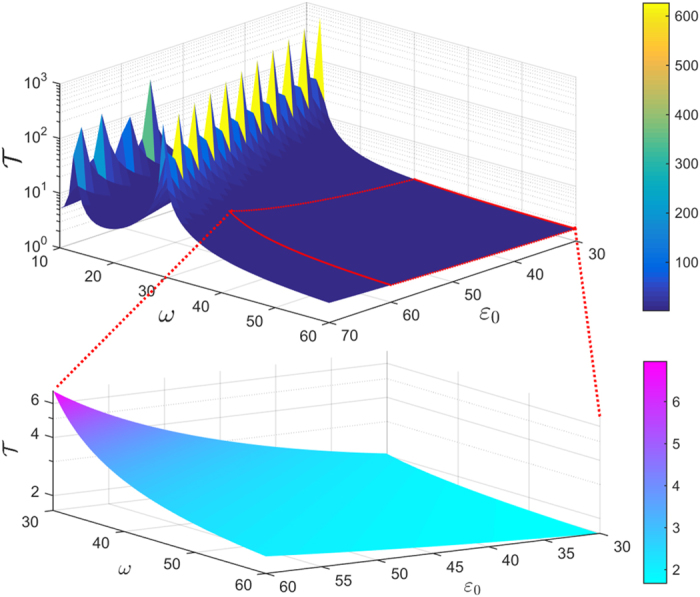
The total operation time 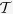 versus the amplitude 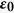 and the frequency 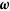 of the driving field.

Figure 4 depicts the relation between the total operation time and the amplitude as well as the frequency of the driving field. It suggests that one should avoid the parameter regions where the coherent destruction of tunneling occurs, since it would take a long operation time to realize the operation 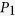 if the destruction happens. Outside these parameter regions, the total operation time can be regulated within a range. Besides, one can readily find in Eq. (5) that the fidelity to realize 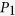 is


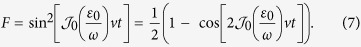


**Figure 5:**
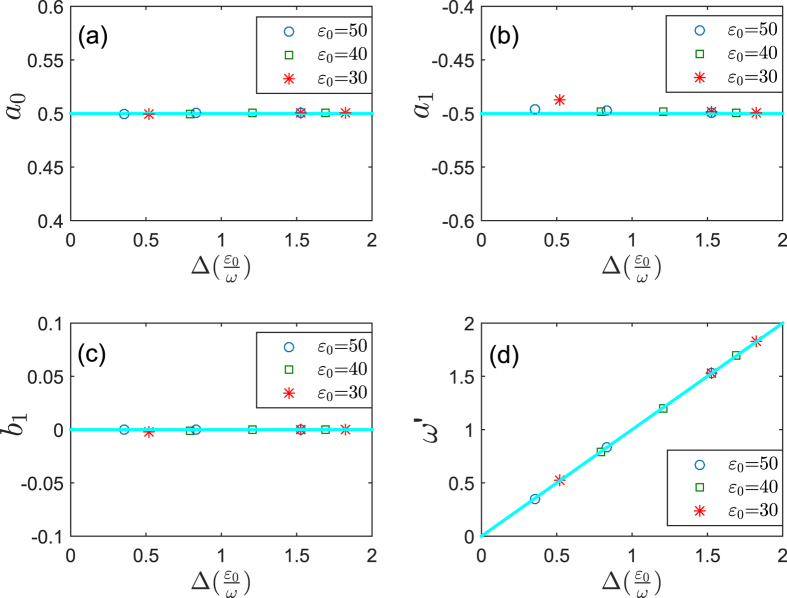
The coefficients (**a**) *a*_0_, (**b**) *a*_1_, (**c**) *b*_1_, and (**d**) 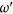 in the fidelity expression versus the quasi-energies gap. The fidelity expression by curve-fitting in MATLAB is given by 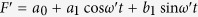. The lines represent the perturbation results given by Eq. (7), while the circles, squares, and stars represent exact results obtained by curve-fitting. Note that the curve-fitting has high degree of precision for the exact results since the values of *R-square* and *Adjusted R-square* approach 1 (≥99.37%) in MATLAB.

We plot the coefficients in the fidelity as a function of the quasi-energies gap by exact and perturbative calculations in Fig. 5. It demonstrates that the perturbation results work very well in the high-frequency limit.

**Figure 6:**
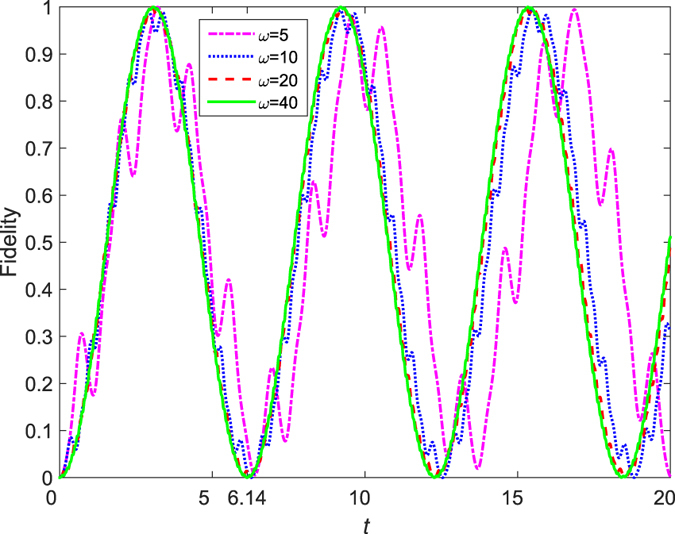
The evolution of fidelity with different frequencies of driving field. We have set the quasi-energies gap 
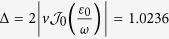
, thus the “period” of the system dynamics is about 
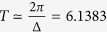
 in the high-frequency limit, which is confirmed by the green solid line and red dash line in this figure.

In order to check the validity of perturbation theory, we plot the system dynamics with different driving frequencies in Fig. 6. We observe that the dynamics is in excellent agreement with the results by perturbation theory when 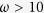 (in units of *v*), while it deviates seriously from the perturbation results when 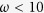 (see the pink dot-dash line in Fig. 6). This suggests that the perturbation theory can be safely used when the frequency of the driving field is at least an order of magnitude larger than the tunnel coupling.

**Figure 7:**
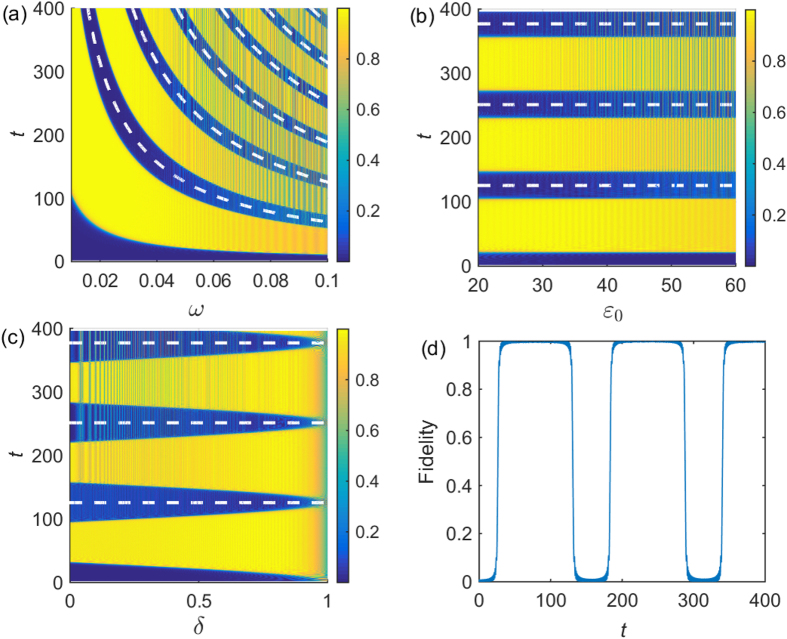
Fidelity as a function of time and different parameters of driving field. The form of driving field takes 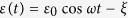, where 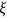 is the offset energy of the driving field. (**a**) 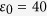, 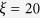. (**b**) 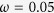, 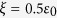. (**c**) 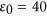, 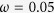, 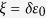. (**d**) 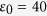, 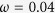, 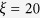. The dash lines are plotted at 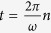, where *n* is an integer running number.

Since the perturbation theory is invalid in the low-frequency limit, one might ask how the system behaves in this limit. If the driving frequency is sufficiently small, the adiabatic condition holds in that the on-site energy changes slowly. We expect that system dynamics (e.g., the fidelity) would manifest periodically at long time-scale (the “period” 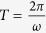). Figure 7 demonstrates the evolution with different parameters of the driving field, where the periodic dash lines verifies our expectation. Besides, one can observe in Fig. 7(a) that high fidelity lasts long time within a period when the frequency of driving field is small, and the fidelity dramatically changes when the frequency of driving field is large (i.e., the yellow lines and blue lines alter frequently). The system dynamics becomes considerably complicated when the amplitude of driving field is large, as shown in Fig. 7(b,c) illustrates how the offset energy of quantum dot affects the system dynamics. Interestingly, high fidelity lasts longer time (see the yellow region) when the offset energy of quantum dot is larger. Note that if the offset energy of quantum dot is sufficiently large, e.g., 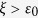, the system dynamics would halt since the value of on-site energy 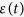 is always positive. Figure 7(d) shows the fidelity as a function of time, indicating that the operation time is about 40 and the time with high fidelity lasts about 80.

### Square-well driving

The periodic square-well field is another waveform that can be easily achieved in practice. In fact these driving fields have already been studied extensively in time-periodic driving system. Recently, it has been shown in experiment^54^ that the Stückelberg interference occurs in a superconducting qubit driven by square-well field. The square-well driving for the on-site energy of the quantum dot can be expressed as,


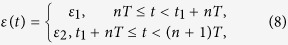


where 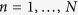 and 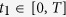. *T* is the period of square-well driving and *N* is the number of evolution period. To realize the operation *P*_1_, the form of square-well field should satisfy (see Methods)


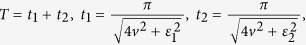



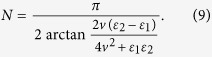


According to Eq. (9) the number of evolution period *N* is not an integer in general. Nevertheless it does not affect the results much because we can take an integer nearest to *N* (the fidelity increases slowly when it approaches 1). In turn, if one designates the period *T* and the number of evolution period *N* of the driving system, the values of on-site energy 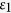 and 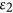 can be resolved by Eq. (9) as well.

**Figure 8:**
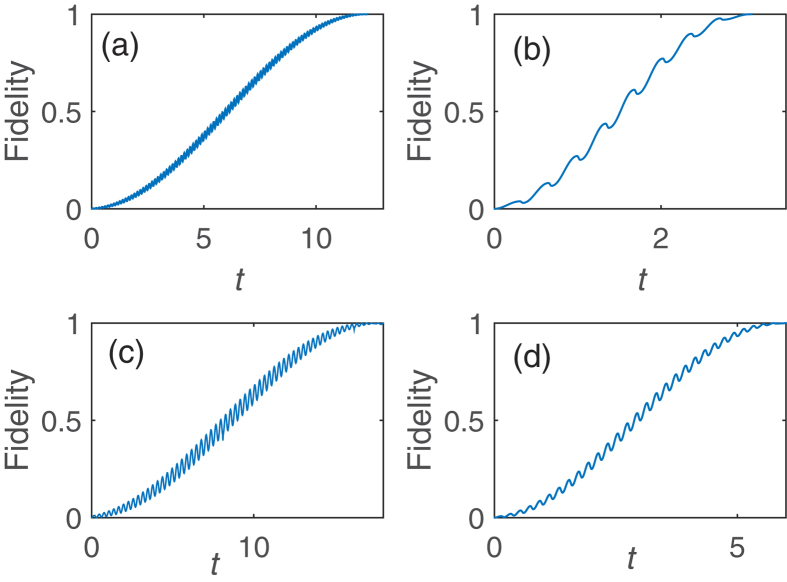
The system dynamics with different forms of square-well driving. (**a**) 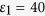, 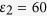. (**b**) 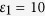, 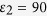. (**c**) 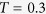, 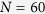. (**d**) 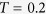, 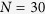. The other two parameters are calculated by Eqs (9) and (29).

Figure 8 show the dynamics of the system with different parameters, where Fig. 8 (a,b) are for different 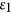 and 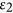 while Fig. 8 (c,d) are for different *T* and *N*. As expected, *P*_1_ can also be realized in the system driven by a square-well field, and the operation time would be shorter if the difference between 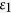 and 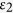 gets larger.

### *δ*-kick

When the on-site energy 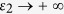, one readily finds in Eq. (9) that 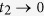. Then the square-well field reduces to a periodic *δ*-kick, i.e.,


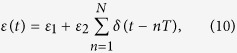


where *T* is the period of *δ*-kick calculated via 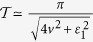. The total operation time for realizing the operation *P*_1_ becomes,


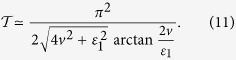


Note that the dynamic behavior of system with the *δ*-kick is quite different from that without it. This can be confirmed by examining a case that the system is under a static driving field 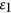, where the evolution operator *U* reads,


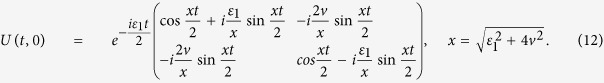


The fidelity to realize the operation *P*_1_ takes 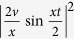, yielding the maximum of fidelity 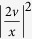. One easily observes that it cannot obtain the operation *P*_1_ when 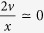, i.e., the on-site energy 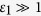. However the situation changes in the presence of *δ*-kick, where the operation *P*_1_ can be achieved regardless of the value of the on-site energy 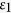 (the value of 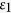 determines the driving period and operation time). The advantage in this case is that the system is still in a stationary state when we remove the *δ*-kick after the operation *P*_1_ is completed.

### Application to other systems

**Figure 9:**
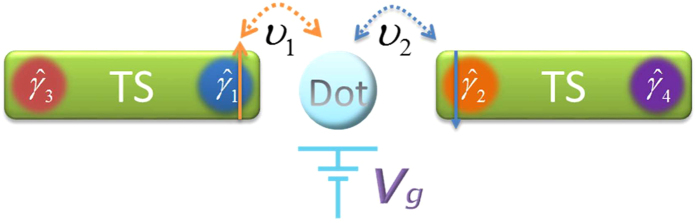
The schematic setup to entangle a conventional qubit and topological qubit.

Not restricted to the above concrete system, the periodic driving method can be widely applied to the other structure of quantum systems. Here we apply it to a system described by the following Hamiltonian^56^, where two TS nanowires couple to a common quantum dot, as illustrated in Fig. 9,


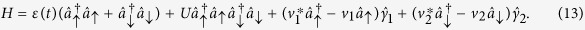



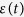
 is the on-site energy of the quantum dot. *v*_*i*_(*i* = 1, 2) denotes the tunnel coupling between the quantum dot and the Majorana mode 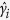. In particular, the spin-up (labeled as 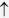) and spin-down (labeled as 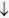) electrons can only tunnel into the Majorana mode 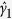 and 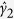, respectively. *U* represents the energy contributed by double occupation on the same quantum dot. When *U* is sufficiently large, the quantum dot is in the Coulomb block regime where it can only hold one electron.

Since the total parity (the electrons in quantum dot and the ordinary fermions formed by Majorana modes) of the hybrid system is conserved, we restrict ourself in the even-parity subspace spanned by 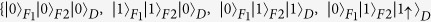, 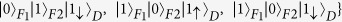, where the subscript *F*_*i*_(*i* = 1, 2) represents the ordinary fermions formed by Majorana modes. The Hamiltonian then can be written as,


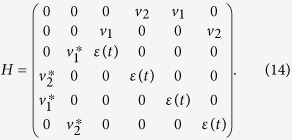


**Figure 10:**
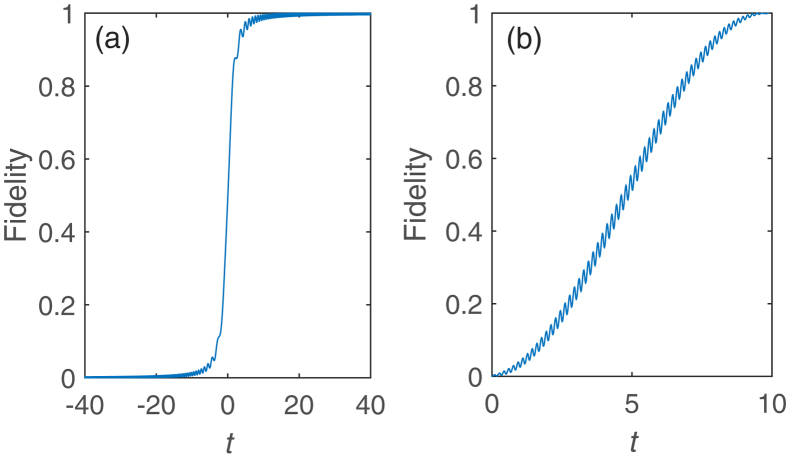
Realizing the operation *P*_2_ by (**a**) adiabatic evolution and (**b**) by periodic square-well driving. The on-site energy of quantum dot is 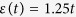 in panel (**a**), and 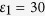, 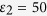 in panel (**b**). 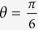, 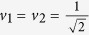. All parameters are in units of *v*.

This quantum dot-Majorana system^55^,^56^ can be exploited to prepare entanglement between spin and topological qubits or quantum information transfer between spin and topological qubits (even for quantum logic gates) by adiabatic evolution. We take the preparation of entanglement (denoting as the operation *P*_2_) between the electron spin and Majorana modes as an example to exemplify how to manipulate the operation time by periodic square-well driving given by Eq. (8). The operation *P*_2_ reads,


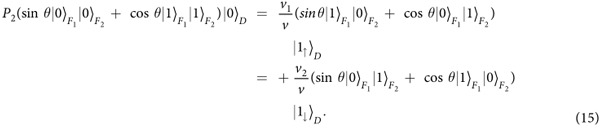


where 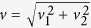. As the Hamiltonian is a 6 × 6 matrix, the analytical expression of evolution operator 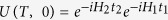 is involved. Here we only give the equations that determine the period of driving field and the total number of evolution periods, i.e.,


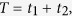



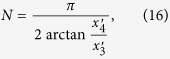


where 
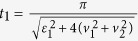
, 
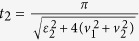
, 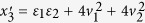, and 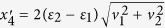. Figure 10 plots the fidelity to realize the operation *P*_2_ as a function of time by adiabatic evolution and periodic square-well driving, respectively. Again, the operation time for adiabatic evolution requires relatively long time since it must satisfy adiabatic condition while the operation time and the period can be regulated in square-well driving.

## Discussions

Until now, we have studied how to implement the operation *P*_1_ by periodically modulating the on-site energy of the quantum dot. For a single operation *P*_1_, it is far from sufficient to permit quantum computation. We next briefly discuss how to realize an arbitrary rotation for a Majorana based qubit by successively executing the operation *P*_1_ twice. As shown in Fig. 1(b), the system Hamiltonian reads


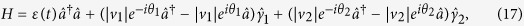


where we have introduced a phase 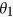 (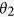) into the tunnel coupling *v*_1_ (*v*_2_). Defining the operator 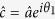, 
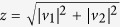
, 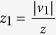, and 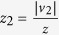, the Hamiltonian (17) becomes,


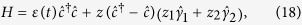


where the phase difference 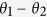 (it can be modulated by the magnetic flux 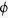) equals 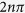, *n* = 1, 2…. The form of Eq. (18) is the same as Eq. (2) if we redefine a new Majorana mode 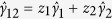, where the tunnel coupling is denoted by *z*. Clearly, the operation 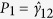 in this notation. For a two level system spanned by 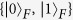, we can express the Majorana operators in terms of Pauli matrices 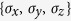, i.e., 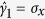, 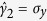, 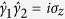. By successively executing the operation 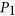 twice with different relative tunnel strengthes, the total operation becomes 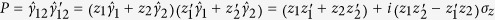, which is exactly an arbitrary rotation around the *z*-axis.

Due to the conservation of total parity, a qubit shall be encoded by four Majorana modes^57^. The Majorana based qubit can be realized by the extended model in Fig. 1(b), where the system consists of three quantum dots coupling to four Majorana modes (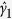, 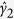, 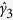, 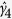) in the TS nanowire with comb structure. In the even-parity subspace spanned by 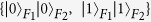, , the operation 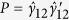 is in fact the rotation around the *z*-axis, and the operation 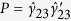 is the rotation around the *x*-axis (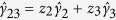), where the ordinary fermion *F*_1_ (*F*_2_) is formed by the Majorana modes 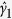 and 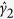 (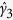 and 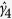).

Finally, we give a brief discussion on the experimental feasibility of our proposal. In order to avoid quasi-particle excitations in the TS nanowire and two electrons occupying the same quantum dot, the superconducting gap and the Coulomb interaction *U* are required to be much larger than the tunnel strength *λ*. For most quantum dot setups the Coulomb interaction 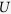 can arrive at the order of meV. Recent experiments^17^,^18^,^58^,^59^ shows that it is sufficient to make the superconducting gap in the order of 0.1–1 meV and the tunnel strength in the order of 1−10 *μ *eV. Considering electron tunnel from an external environment, the decoherence time of Majorana based qubit is in order of 10 ns–0.1 ms^49^. Besides, due to the electron-phonon interaction the lifetime of quantum dot is in order of 16 ns^60^,^61^. When we take the tunnel strength in the order of 1−10 *μ *eV, the total operation time by periodic driving can reach less than 2 ns, which is much smaller than the system decoherence time. Note that the tunnel coupling between the quantum dot and the Majorana mode depends on both differences among the on-site energies and the tunnel barriers. By making use of periodic driving on the on-site energy of quantum dot, the tunnel coupling would change consequently. Noting that we can employ additional electrostatic gates to manipulate the tunnel barriers, the tunnel coupling can still maintain constant even the on-site energy of quantum dot changes. Indeed, the possibility of controlling the tunnel coupling in semiconductor nanowire has been shown experimentally^62^. So the voltage of electrostatic gates can be manipulated periodicity to make the tunnel rate remain unchanged in our system, especially for the periodic square-well case (since it has only two different values of on-site energy).

In summary, we have presented a scheme to realize quantum operation (quantum gates) by periodic driving. The operation time can be exactly controlled by modulating the amplitude or frequency of the driving field. By solving the time-dependent Schrödinger equation with perturbation expansion in the high-frequency limit, we have given an expression for the quasi-energies gap in the sinusoidal driving and found that the total operation time can be manipulated by designing the amplitude and frequency of the driving field. In the low-frequency limit, due to the invalidity of perturbation theory, we study the system dynamics by numerical simulations. The results are almost consistent with those given by adiabatic evolution. Different from adiabatic evolution, the system in the low-frequency limit manifests more intricate behaviors and the operation time can also be regulated by the driving field. In particular, the time that the high fidelity lasts are closely related to the frequency and offset energy of the driving field. With the periodic square-well driving, we have derived an analytical expression for the evolution operator without any approximations. By this expression, we can calculate the amplitude of the square-well driving under fixed operation time and period of the driving field. We have also discussed realization of quantum operations by a *δ*-kick, which can be treated as a deformed square-well driving. Besides, the periodic driving method can be applied to the other quantum systems-it opens up a new avenue to manipulate the operation time in the topological quantum computation.

## Methods

### Floquet theory

Assume that the system Hamiltonian is periodic in time, i.e., 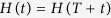, where *T* is the period and the driving frequency 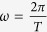. The Floquet theory^63^ tells that the solutions of Schrödinger equation have the following form 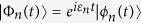, where 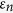 is the quasi-energy and the Floquet state 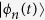 satisfies 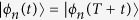. At the same time the eigenvalue equation of the system is (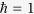)


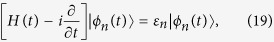


where 
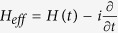
 is the so-called Floquet operator. To solve this equation it is very instructive to introduce an extend Hilbert space^64^ consisting of time-periodic functions with the inner product defined by 
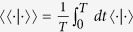
. We will solve the eigenvalues and eigenstates of the Floquet operator by perturbation theory in the following.

### Perturbation theory

With sinusoidal driving, due to the total parity conservation, it is convenient to study in the even parity (or odd parity) subspace. Then the Hamiltonian can be expressed by a 2 × 2 matrix, which can be divided into two parts: the on-site energy term 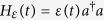 and the tunnel term 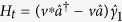. In this following, we assume that the tunnel term is a perturbation^65^,^66^. Since 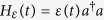 is diagonal in the space spanned by 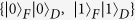, when substituting into Eq. (19), the eigenstates of 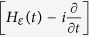 are given by


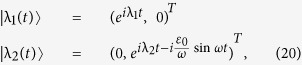


where 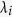 (*i* = 1, 2) is the corresponding eigenvalue (i.e., quasi-energy). In consideration of the periodicity of the Floquet states, two quasi-energies are zero (modulo 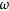) in the zeroth-order approximation. As a result the two time-dependent eigenstates can be approximately viewed as time-independent eigenstates in the high-frequency limit (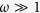), i.e., 
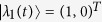
 and 
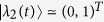
. The quasi-energies in the first-order approximation can be obtained by diagonalizing the matrix^66^


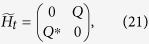


where the matrix element are calculated by 
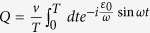
. This gives the quasi-energies 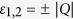 (in the “first Brillouin zone”) and the corresponding eigenstates 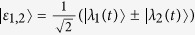. The quasi-energies gap is consequently given by 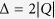. In the light of the identity


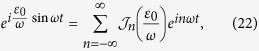


where 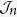 is the 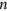-order Bessel function, we can finally obtain the analytical expression of quasi-energies gap in the maintext: 
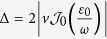
.

### The derivation of Eq. (9)

With square-well driving, the on-site energy can be written as,


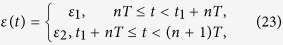


where *n* = 1, 2, 3… and 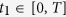. The evolution operator *U* within one period of time takes 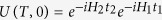 with 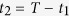. After some tedious but straightforward algebra, one can obtain,


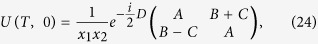


where


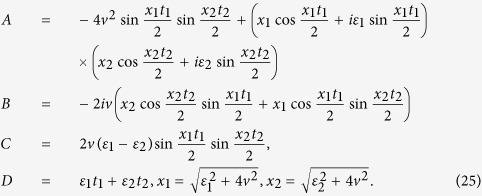


At first, we design the driving time *t*_1_ (*t*_2_) for the on-site energy 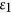 (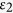) to satisfy 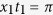 (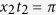), that is,


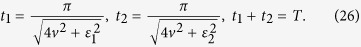


Certainly, the period of the square-well driving is fixed. According to Eq. (26), the evolution operator can be further simplified, i.e.,


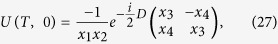


where 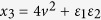 and 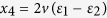. After *N* periods of time, the final evolution operator becomes


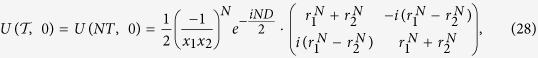


where 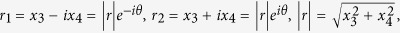 and 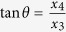. Clearly, it requires 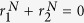 in order to realize the operation *P*_1_ perfectly (up to a global phase factor). By making the vectors 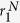 and 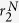 produce a *π*-phase difference, that is, 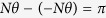, one can readily obtain the number of evolution periods,


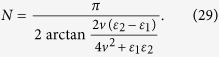


## Additional Information

**How to cite this article**: Shi, Z. C. *et al.* Quantum gates by periodic driving. *Sci. Rep.* **6**, 22077; doi: 10.1038/srep22077 (2016).
